# Turnover intention and its antecedents: The mediating role of work–life balance and the moderating role of job opportunity

**DOI:** 10.3389/fpsyg.2023.1137945

**Published:** 2023-04-03

**Authors:** Roselina Ahmad Saufi, Samsidine Aidara, Noorshella Binti Che Nawi, P. Yukthamarani Permarupan, Noor Raihani Binti Zainol, Abdul Samad Kakar

**Affiliations:** ^1^Malaysian Graduate School of Entrepreneurship and Business, Universiti Malaysia Kelantan, Kota Bharu, Malaysia; ^2^Faculty of Entrepreneurship and Business, Universiti Malaysia Kelantan, Kota Bharu, Malaysia; ^3^Department of Management Sciences, University of Loralai, Loralai, Pakistan

**Keywords:** occupational prestige, organisational reputation, job opportunity, work–life balance, human resource management practises, turnover intention, PLS-SEM

## Abstract

Due to the higher attrition rate in higher education institutions (HEIs), the attraction and retention of top talents in higher education have become a challenge for human resource (HR) professionals. The primary area of discussion among business executives and HR professionals is how top talent can be retained and maintained. Thus, the purpose of this study is to examine the impact of human resource management practises (HRMPs), oraganisational reputation (OGR), occupational prestige (OPP) and work–life balance (WLB) on turnover intention (TOI) of the academics working in HEIs. The study also aims to examine WLB as a mediator and job opportunity (JBO) as a moderator of the relationships mentioned above. Data collected through an online survey from 466 respondents were analysed using partial least square structural equation modelling. The findings of the study showed that OGR, OPP and WLB were negatively related to TOI. However, the impact of HRMPs on TOI was not direct; instead, it was mediated by WLB. The findings also demonstrated that WLB significantly mediated the relationship between OGR and OPP. Furthermore, the results also confirmed that JBO significantly moderated the relationship between WLB and TOI. The findings of the study provide guidelines for a comprehensive retention strategy and a holistic model of academics TOI that can assist HR professionals, policymakers and management in developing an effective strategic recruitment and retention plan.

## 1. Introduction

The dynamic, unstable and unpredictable competitive landscape necessitates that organisations make successful and efficient use of their physical, financial, legal and human resources in order to surpass competitors (Gulati and Monica., 2016). Human resources (employees) are organisations’ most precious and unique resources and the unrivalled source of their competitive advantage (Putra and Cho, 2019). Employees who are completely devoted and satisfied and stay with the organisation for a longer time are crucial for attaining organisational goals in a dynamic and complicated environment (Silva and Dias, 2023). Organisations use human resource management practises to recruit, inspire and keep employees. However, retaining employees and encouraging them to feel a strong loyalty to the organisation are significant challenges currently confronting all organisations. High turnover intention (TOI) inhibited primary business goals and resulted in substantial organisational losses (Kumar et al., 2021). The loss of a highly competent employee affects organisational reputation, profitability and performance (Muzaffar and Javed, 2021; Kakar et al., 2023), commitment and overall vision (Kumar et al., 2021). Additionally, the loss of competent academicians could negatively affect the reputation of a university and the quality of work produced.

Thus, due to the rapidly changing environment and growing trend of global competitiveness, maintaining talented personnel determines business success or failure (Sepahvand and Khodashahri, 2021). Employees are the major asset of a university and the incomparable source of its competitive advantage (Samad and Saufi, 2017). Compared to other sectors, HEIs rely on academic employees’ abilities and dedication (Robyn and Du Preez, 2013). The global business services forum (2017) reported that education sector HR practises must adapt to the reality that the average employee tenure is approximately 2.5 years. Thus, the lack of a retention strategy can affect a university’s performance and governmental efforts to reform education. To minimise attrition rates and retain talented and skilled employees, universities have adopted a range of human resource management practises (HRMPs; Rathi and Lee, 2016), such as involving employees in decision-making (Ji-Young and Huang, 2021), providing sufficient development and advancement opportunities (Rashid, 2021), sustaining work–life balance (Aman-Ullah et al., 2022), ensuring appropriate pay and organisational reputation (OGR; Deniz, 2020; Kakar et al., 2021) and incorporating health and safety programmes. Thus, implementing these human resource policies not only improved employee performance and loyalty to the institution (Muzaffar and Javed, 2021), but also decreased their TOI and actual turnover (Rathi and Lee, 2016). However, most studies on employees’ TOI are at the organisational level, which has overlooked personal or employee opinions and perceptions (Kakar et al., 2018). Thus, this study’s first objectives are to examine to what extent employees’ perceptions of HRMPs, OGR, OPP and work–life balance (WLB) are significant in their decision to quit or not quit a job.

Furthermore, a review of earlier studies revealed a lack of research regarding the potential mediating role of WLB on the relationship between HRMPs, OGR, OPP and employee TOI, specifically in academia. Although it is commonly recognised that WLB significantly influences both employees retention and satisfaction (Kakar et al., 2019; Aman-Ullah et al., 2022), the current literature does not provide a clear understanding of the underlying mediating mechanism of WLB between TOI and TOI determinants (Kakar et al., 2021). Thus, additional studies on the mediating mechanism are required to better understand how HRMPs, OGR and OPP are related to TOI (Kakar et al., 2022). Scholars also argue that there are a number of variables that can mediate the relationship of TOI with its predictors (Kakar et al., 2019; Kakar et al., 2022). Therefore, keeping in view the call of researchers, this study adds to the literature by investigating WLB as a mediator between HRMPs, OGR, OPP and TOI so that the necessary action may be taken to reduce academic TOI.

In addition, most of the research has overlooked the impact of factors that are beyond the control of the organisation (Kakar et al., 2023). For instance, job opportunity (JBO) is an external aspect that is potentially crucial in comprehending employees’ attitudes and behaviours (Kakar et al., 2018). This argumentation is supported by the observation that more work options are available to employees when JBOs are higher, which increases employees’ propensity to leave their jobs. Thus, we predict that the relationship between WLB and employee TOI is moderated by job opportunities.

In sum, this study adds to the literature by examining the impact of HRMPs, OGR and OPP on employee TOI and WLB of employees working in Malaysia. The second contribution of this study is examining the mediating role of WLB between HRMPs, OGR, OPP and employee TOI. One significant contribution of the study is investigating job opportunities as a moderator of the relationship between WLB and TOI. Thus, understanding the mediating and moderating mechanisms of WLB and JBO improve policymakers’ comprehension, capability and flexibility to formulate and implement strategic policies that satisfy academic demands and preferences and job challenge resolution capacity. Thus, in addition to the theoretical contributions, the findings provide important practical and managerial contributions to the literature.

## 2. Literature review

### 2.1. Theoretical foundation

The theoretical foundation of this study is based on the theory of planned behaviour (TPB; Ajzen, 1991) and social exchange theory (SET), which Blau developed in 1964 (Blau, 1964). The TPB states that intention is the primary prerequisite of actual action (Ajzen, 1991), and an employee’s TOI significantly affect their actual behaviour (Samad and Saufi, 2017). The theory also demonstrates employees’ attitudes, subjective norms and perceptions of their ability to control their behaviour affect their TOI. A person’s attitude is defined as their favourable or unfavourable evaluation of a particular behaviour (Ajzen, 1991). Employees are more likely to establish a positive intention to engage in a behaviour if they have a positive attitude towards it. Therefore, employees are more inclined to remain on the job when they have a positive attitude towards the organisation or vice versa. Additionally, employees are more inclined to remain with an institution if they perceive that outsiders have favourable impressions of its reputation and occupational prestige (i.e. positive subjective norms). Moreover, the theory also posits that employees are free to select from diverse available and accessible jobs. According to Kakar et al. (2019) and Rashid (2021), employees’ propensity to leave their jobs increases when JBOs are plentiful, as they can quickly locate a promising job.

The SET states that social and economic interactions are essential in the employer-employee relationship (Kakar et al., 2021). For instance, employees expect social and material exchanges from their company that are a part of organisational practises in a working relationship (Kakar et al., 2019). Conversely, the social connections that a business organisation expects from its employees is to accept the organisational policies, philosophies, missions, goals, values and intent to stay with the organisation. According to Kakar et al. (2019), employees who recognise that the organisation provides HRMPs will feel appreciated by the organisation and reciprocate this by exhibiting a positive work attitude and fewer plans to leave. Furthermore, employees who feel that organisational practises are prevalent are more likely to understand organisational principles and values, increasing their organisational fit, commitment and satisfaction and decreasing their desire to leave their jobs (Kakar et al., 2022). Thus, this study uses the TPB and SET as theoretical foundations to ascertain academic employees’ desire to leave their jobs.

### 2.2. Hypothesis development

#### 2.2.1. Turnover intention

**Figure 1:**
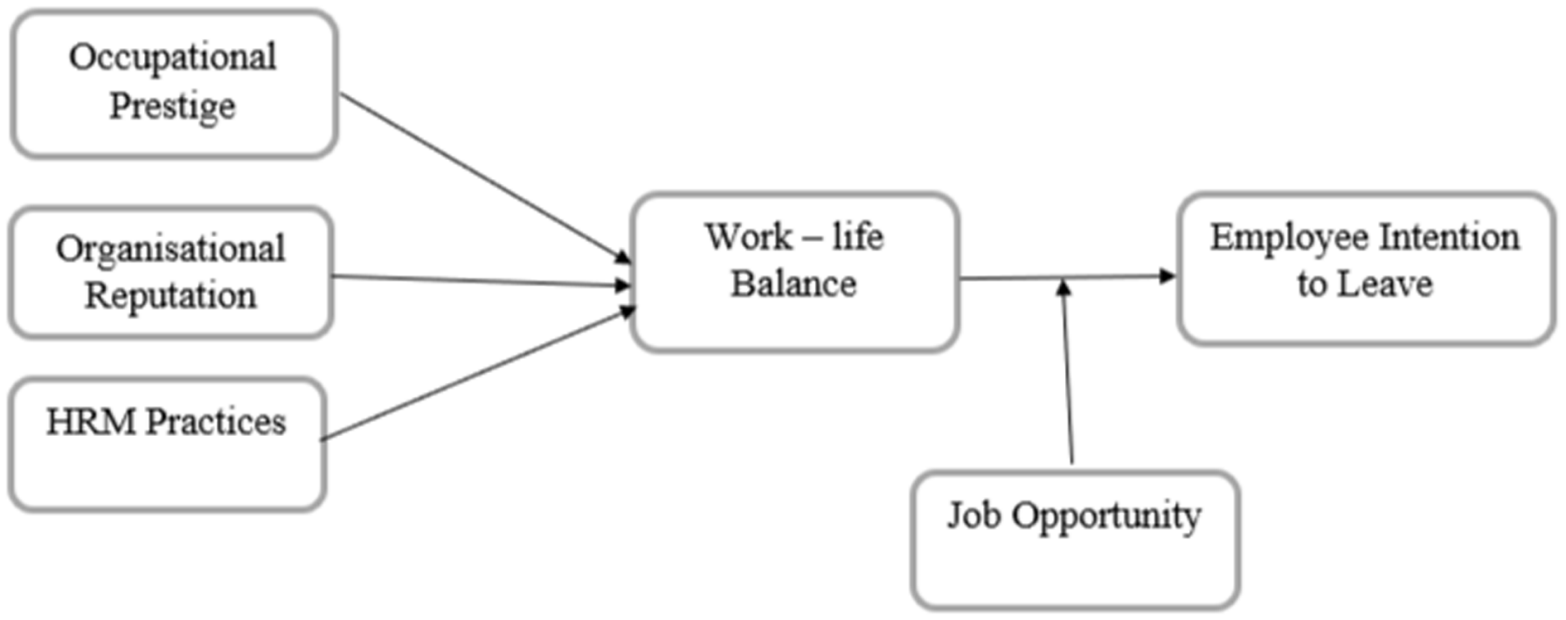
Research model.

Turnover intention refers to the desire to relocate or leave an organisation to find a better job (Lestari and Margarethaa, 2021), and it is the most important indicator of actual leaving behaviour (Ajzen, 1991). An employee’s intention to leave is the final step of leaving the organisation, whether through resignation or termination (Kakar et al., 2021). Despite the plethora of turnover research, academicians remain divided on how to operationalise different types of turnover and how they are related to one another. In contrast, employee retention, which is the opposite of TOI, is a challenging endeavour that necessitates the reduction of both actual turnover and their eagerness to leave the organisation. Employee retention is currently the most significant issue facing business executives, which results in a shortage of skilled workers, slow economic growth and high employee turnover (Sepahvand and Khodashahri, 2021). Therefore, organisations should recognise valuable and productive employees and satisfy their needs at the workplace, family and other needs such as education and social interaction. The satisfaction of various employees’ needs will result in the reduction of employees’ TOI. Figure 1 illustrates the research model of the study.

#### 2.2.2. Occupational prestige

The concept of occupational prestige (OPP) refers to employees’ perceptions of how the public perceives their occupation (Zhu et al., 2020). The OPP is also defined as organisational members’ opinions and perceptions of how outsiders assess their organisational position and reputation (Mael and Ashforth, 1992; Rathi and Lee, 2016), and it is also known as an employee’s perception of the organisational status among others (Dalton et al., 2020). A high level of professional prestige naturally enhances employees’ self-esteem and aids their success primarily through job satisfaction (Sadeghnezhad and Allhosseini, 2019). When employees believe their organisation is highly esteemed, quitting the organisation becomes expensive for them because it would mean losing a significant source of self-worth and self-esteem (Guerrero and Challiol-Jeanblanc, 2017). If an employee believes that their working relationships are detrimental to having a positive self-image and the provision of work-life balance, their attitudes and behaviours at work will deteriorate (Bright, 2020). Furthermore, with greater OPP levels, the employer is likely to increase an employee’s desire for self-improvement and self-esteem, which in turn positively affect job satisfaction and negatively influence burnout and TOI (Dalton et al., 2020). For public and private universities, a prestigious image will enable them to establish higher tuition fees, attract and retain talented employees and enrol excellent students. Additionally, strong perceived external prestige increases employee engagement and organisational affiliation (Rathi and Lee, 2016). Recent empirical studies demonstrated that OPP was positively related to job satisfaction (Dalton et al., 2020), negatively associated with TOI (Zhu et al., 2020) and indirectly, with organisational identification (Zhu et al., 2020) and affective commitment (Rathi and Lee, 2016). The TPB also explains the impact of OPP on TOI and WLB. This theory posits that employees’ intention is the product of their attitude towards a behaviour. For instance, if employees’ attitude is positive towards a behaviour, they are likely to engage in that behaviour and vice versa. Following TPB, we argue that OPP also reflects employees perceptions towards the occupation. If employees’ perception of prestige is high, they are more likely to stay with their job and experience better WLB. Thus, the following hypotheses (H) are formulated:

*H1A:* Occupational prestige negatively affects turnover intention.

*H2A:* Occupational prestige positively affects work–life balance.

#### 2.2.3. Human resource management practises

The primary goal of strategic HRM is to develop strategic organisational competence by attracting talented, loyal and ambitious personnel (Sepahvand and Khodashahri, 2021). Managers can easily maintain their staff by implementing strategies based on improving HRM systems. This study predicts that an employee’s intention to leave their employer is influenced by their attitude (perceptions) towards HRMPs and their behaviour (Samad and Saufi, 2017). Perceived HRMPs, on the other hand, refer to employees’ psychological assessments, interpretations and experiences of intended and actual HRMPs (Boxall and Purcell, 2011; Kakar et al., 2022). Thus, an organisation that successfully implements HRM strategies (training and development opportunities, compensation system and performance management) might create a positive image or perception of the organisation, which in turn would reduce its TOI. Besides, employees are more inclined to reciprocate a variety of successful job outcomes when they believe their employer is investing in them by providing HRM practises (Kakar et al., 2018). Therefore, employees with a favourable impression of HRM practises demonstrated increased commitment to the organisation and job satisfaction (Muzaffar and Javed, 2021), well-being, WLB (Hasan et al., 2021) and intention to stay (Ozioma et al., 2022). Thus, based on the TPB and related literature, the following hypotheses are formulated:

*H1B:* HRMPs negatively affect turnover intention.

*H2B:* HRMPs positively affect work–life balance.

#### 2.2.4. Organisational reputation

The organisational reputation (OGR) is one of the most critical and strategic resources developed over time, and is associated with diverse successful results for employees and the company (Kakar et al., 2021). The OGR refers to employees’ perception of organisational past performance and potential future and reflects organisational business attractiveness to important constituencies compared to other top competitors (Al Haraisa and Al-Haraizah, 2021). The organisation’s reputation is also defined as the reflection of its prior decisions and actions (Nguyen and LeBlanc, 2018). Deniz (2020) stated that OGR is based on employees’ perceptions and evaluations, which are based on direct interactions between stakeholders and the company, employees or other organisational representatives. A strong reputation helps in the attraction and retention of talented employees and provide a competitive edge (Zhu et al., 2020), which improves organisational financial performance and profits (Kakar et al., 2021). Furthermore, OGR influences employees’ attitudes and behaviour (Deniz, 2020), whereas a negative OGR perception may have a negative impact employees’ attitudes and behaviour. Additionally, workers in companies with poor reputations could exhibit undesirable attitudes and actions (Al Haraisa and Al-Haraizah, 2021), which include lower motivation, productivity and performance levels (Deniz, 2020). Empirical investigations determined that employees often leave companies with poor reputations (Deniz, 2020; Kakar et al., 2021). Thus, based on the above-mentioned discussion, the following hypotheses are proposed:

*H1C:* Organisational reputation negatively affects turnover intention.

*H2C:* Organisational reputation positively affects turnover intention.

#### 2.2.5. Work–life balance

Work–life balance is defined as the degree to which an employee is consistently satisfied with their family, job and personal roles (Adkins and Premeaux, 2019; Kakar et al., 2022). One of the main obstacles in the current dynamic and competitive environment is creating a work-life and family-life balance because they are interconnected. Employees who successfully manage their personal and professional lives report higher job satisfaction, which reduces their potential to leave the job (Hasan et al., 2021). Furthermore, employees who can minimise work–family conflict (Zheng et al., 2016) gain a higher WLB level, improved health and well-being (Aman-Ullah et al., 2022) and diminished intention to leave. A lack of WLB practises, such as prolonged working hours, limits employees’ time for social and familial activities, detrimentally affects WLB and leads to stress and a desire to leave their job (Kakar et al., 2019). Recent empirical studies confirmed that WLB negatively affected TOI (Lestari and Margarethaa, 2021) and positively affected employee retention (Ozioma et al., 2022). Based on the related literature and SET, we argue that if employees have a balance between work and family life, they are more likely to feel satisfied and happy with work and family responsibilities, which, in the norms of reciprocity, will reduce their TOI. Thus, we hypothesise that:

*H3:* Work–life balance negatively affects turnover intention.

#### 2.2.6. The mediating effect of WLB

As stated above, WLB has significant influences on an organisation’s performance as well as has a strong correlation with employees’ cognitive capacity, psychological well-being and emotional balance (Lestari and Margarethaa, 2021). Organisations implement WLB strategies to reduce stress and work–family conflict (Kakar et al., 2022) and eventually increase employee work performance, organisational commitment and the desire to stay (Kakar et al., 2021). For employees to achieve a healthy WLB, numerous factors, such as work flexibility and the integration of family-friendly policies and benefits, are critically significant (Santhanam et al., 2021).

Furthermore, empirical studies revealed that WLB is a significant predictor of employee well-being and job commitment (Lestari and Margarethaa, 2021), productivity and performance (Aman-Ullah et al., 2022), which are essential for long-term organisational viability and growth (Ang, 2020). While the literature highlighted the significance of WLB practises, few studies examined WLB practises from the employees’ perspectives (Kakar et al., 2021; Ozioma et al., 2022). More importantly, very limited studies have investigated WLB as a mediating variable between OPP, OGR, HRMPs and TOI. This study bridges this gap in the existing literature by investigating the mediating role of WLB on the association between TOI, OPP, OGR and HRMPs. Based on TPB and SET, we argue that when employees believe that they work in an occupation and organisation that is highly prestigious and reputed, they are more likely to feel pride at the workplace and at home. As a result, they will experience reduced work–family conflict and WLB. This perception of WLB, in turn, reduces their TOI. Besides, when an organisation invests in employees by providing HRMPs that are beneficial for their work and family life, they will experience WLB and this perception of WLB will reduce their TOI. Thus, based on TPB, we hypothesise that:

*H4A:* Work–life balance mediates the relationship between OPP and turnover intention.

*H4B:* Work–life balance mediates the relationship between HRMPs and turnover intention.

*H4C:* Work–life balance mediates the relationship between OGR and turnover intention.

#### 2.2.7. The moderating effect of job opportunities

Job opportunity refers to the perceived accessibility of jobs outside the organisation (Price and Mueller, 1981). The JBO demonstrates an employee’s conviction that a promising and profitable career is available outside the company (Gainau, 2021). Kakar et al. (2018) stated that JBO is a potential external and contextual factor that may be essential to understand how employees’ job-related attitudes and behaviours. Employees have a low propensity to move if they are satisfied in their existing positions or believe that there are few alternatives available in the labour market (Huang et al., 2017). Scholars have found that employees would not leave an organisation until they perceive there are better JBOs for their expertise and skills (Rashid, 2021; Kakar and Saufi, 2021). Employees have more employment possibilities when JBOs are greater, which increases their TOI. Additionally, employees are more inclined to change professions or leave their current positions if they feel that alternative jobs align with their values and ambitions and enhance their WLB. Accordingly, it is expected that JBO not only enhances employees’ TOI but also moderates the link between WLB and TOI. Typically, moderators change the direction or strength of the causal link in either a positive or negative way (Amankwaa and Anku-Tsede, 2016). The availability and accessibility of alternative job opportunities can change the relationship between WLB and employee TOI, either negatively or positively. Prior studies have also found that JBO moderate the impact predictors on TOI (Kakar et al., 2018; Kakar and Saufi, 2021). Thus, the following hypotheses are formulated:

*H5A:* Job opportunity positively affect turnover intention.

*H5B:* Job opportunity will moderate the relationship between WLB and turnover intention.

## 3. Research methodology

### 3.1. Sample size calculation and data collection

The respondents for the study were academic staff members of Malaysian HEIs. This study utilised cross-sectional design, and an online survey was employed to gather data. The respondents were informed about the purpose of the research and the participants’ informed consent was obtained. The study sample size was calculated using G-Power 3.1 with a power of 0.95 and an effect size of 0.15. The required sample size for the model was 146 with six factors (Faul et al., 2007). A minimum threshold of 200 observations was necessary for partial least square structural equation modelling (PLS-SEM; Chin, 2010). Kline (2005) suggested that a sample size >200 should be considered significant for a non-complex model. To avoid potential issues stemming from a small sample size, 466 academic employees of public universities participated in this study.

The ethical statement that is provided at the beginning of the survey states that there is no compensation and no known risk associated with responding. The respondents were also assured of the anonymity and confidentiality of the study. Participation in the survey was voluntary, and we only moved forwards with their consent.

### 3.2. Research instrument

The items used to measure the study variables were obtained from previously established reliable scales. The OPP and OGR (seven and six items, respectively) were adopted from Carmeli and Tishler (2005). The HRMPs were measured with nine items scale adapted from Alfes et al. (2013). The JBO was assessed using four items from Peters et al. (1981) and Daly and Dee (2006), while WLB was assessed with four items adapted from Zheng et al. (2016). The six items scale of TOI were obtained from Kumar et al. (2021) and O’Reilly et al. (1991).

### 3.3. Common method variance

Social science research methodologies are frequently biassed because of data collection from a single source (Podsakoff et al., 2003). Thus, two strategies were used in this study to reduce the influence of common method variance (CMV; Podsakoff et al., 2012). The first method distinguished the measurement item of each independent and dependent variable of the study. Second, the full collinearity of all constructs was tested as recommended by Kock (2015) to identify the CMV. The variance inflation factors (VIFs) for HRMPs (2.856), OGR (3.109), OPP (2.527), WLB (2.756) and JBO (1.451) were all <3.3, which indicated the absence of bias from the single-source data (Podsakoff et al., 2012).

### 3.4. Multivariate normality

The data multivariate normality was evaluated using the WebPower online tool (source: https://webpower.psychstat.org/wiki/tools/index). The data demonstrated a non-normality issue, as demonstrated by the calculated Mardia multivariate skewness and kurtosis coefficient, and value of ps, which were all <0.05 (Cain et al., 2017).

### 3.5. Data analysis

The PLS-SEM was used to test the model and assess the hypotheses. The PLS-SEM for hypotheses evaluation is effective in various studies and widely applied (Hair et al., 2019). The PLS-SEM flexibility in data allocation renders it ideal for complex model analysis. Furthermore, in PLS-SEM, the measurement model is assessed to confirm construct reliability, convergent validity and discriminant validity before the structural model is constructed (Henseler et al., 2017). The internal consistency reliability was determined using Cronbach’s alpha (CA), Dillon-Goldstein’s (DG) rho and composite reliability (CR). Convergent validity was measured using the average variance extracted (AVE; Hair et al., 2019). Discriminant validity was evaluated using the heterotrait-monotrait ratio (HTMT), the Fornell-Larcker criterion and cross-loading. The hypotheses were tested by estimating path coefficients (Beta), confidence interval bias, *t*-value, value of *p*, R^2^ and effect size (Hair et al., 2019).

## 4. Findings

### 4.1. Respondents’ demographic profile

There were similar numbers of male (50.43%) and female (49.57%) respondents (see Table 1) in this study. Most respondents held PhDs (71.03%), followed by respondents with master of philosophy degrees (22.74%) and master degrees (6.23%). Most respondents were Malay (49.14%), followed by Indian (21.67%), Chinese (19.95%) or other ethnicities (9.23%). Up to 30.90% of the respondents had worked for 1–5 years, 55.36% for 6–10 years, 11.80% for 11–15 years and 1.94% for >15 years. A nearly equal proportion of respondents worked in their hometown (49.79%) and away from their hometown (50.21%). More than half of the respondents (52.80%) were between 36 and 45 years old, followed by respondents aged 25–35 (33.90%) and 46–55 years old (13.30%). Most respondents were married (65.66%), while 21.67% were single and 12.66% were divorced. The majority of respondents were senior lecturers (42.49%) followed by those who were lecturers (29.40%), assistant professors (20.39%) or professors (7.72%).

**Table 1:** Respondents’ demographic profile.

	*N*	%		*N*	%
Gender			Institution location		
Male	235	50.43	Hometown	232	49.79
Female	231	49.57	Away from hometown	234	50.21
Total	466	100	Total	466	100
Education level			Age (years)		
Master	29	6.23	25–35	158	33.90
M.Phil	106	22.74	36–45	246	52.80
PhD	331	71.03	46–55	62	13.30
Total	466	100	Total	466	100
Work experience (years)			Marital status		
1–5	144	30.90	Single	101	21.67
6–10	258	55.36	Married	306	65.66
11–15	55	11.80	Divorced	59	12.66
>15	9	1.94	Total	466	100
Total	466	100			
Race			Designation		
Malay	229	49.14	Lecturer	137	29.40
Chinese	93	19.95	Senior lecturer	198	42.49
Indian	101	21.67	Assistant professor	95	20.39
Other	43	9.23	Professor	36	7.72
Total	466	100	Total	466	100

### 4.2. Construct reliability and validity

The results of the measurement model revealed that all constructs CA, DG rho and CR scores were > 0.70 (Hair et al., 2019; see Table 2). Thus, all indicators were considered reliable. The AVE value for all constructs were > 0.50, thereby demonstrating suitable and sufficient convergent validity (Hair et al., 2019). Besides, there was no issue with multi-collinearity among the study constructs, where the VIF for each construct was <3.3 (Hair et al., 2019). Thus, the study constructs fulfilled the reliability and convergent validity requirements.

**Table 2:** Reliability analysis.

Variables	Items	CA	Dg rho	CR	AVE	VIF
HRMP	9	0.974	0.975	0.978	0.831	2.856
OGR	6	0.978	0.978	0.981	0.882	3.109
OPP	7	0.970	0.971	0.975	0.850	2.527
JBO	4	0.987	0.988	0.990	0.963	1.451
WLB	4	0.976	0.976	0.982	0.933	2.756
EIL	6	0.983	0.983	0.986	0.920	

CA, cronbach’s alpha; DG rho, dillon-goldstein’s rho; CR, composite reliability; AVE, average variance extracted; VIF, variance inflation factors. Author’s data analysis.

Furthermore, all HTMT ratio scores were < 0.90, which supported appropriate discriminant validity. In addition, the Fornell-Larcker indicators achieved the discriminant validity criteria (see Table 3). The discriminant validity tested through cross-loading (Appendix A1) suggested that all construct cross-loading values achieved a minimum threshold value of 0.708 (Hair et al., 2019; see Appendix A1). Thus, the findings demonstrated that discriminant validity was fulfilled for the study constructs.

**Table 3:** Discriminant Validities.

	EIL	HRMP	JBO	OGR	OPP	WLB	JBO × WLB
HTMT ratio							
EIL							
HRMP	0.566						
JBO	0.567	0.422					
OGR	0.575	0.771	0.412				
OPP	0.574	0.722	0.404	0.759			
WLB	0.651	0.676	0.509	0.678	0.615		
JBO x WLB	0.455	0.378	0.083	0.324	0.284	0.524	
Fornell-Larcker criterion							
EIL	0.959						
HRMP	−0.555	0.911					
JBO	0.559	−0.415	0.981				
OGR	−0.564	0.754	−0.405	0.939			
OPP	−0.56	0.703	−0.396	0.739	0.922		
WLB	−0.637	0.66	−0.5	0.663	0.599	0.966	

OPP, occupational prestige; HRMP, human resource management practise; OGR, organisation reputation; WLB, work–life balance; JOB, job opportunity; EIL, employee intention to leave.

### 4.3. Path analysis

The results of the structural model revealed that the *R*^2^ value from TOI was 0.569, with the five exogenous constructs indicating that the five constructs (HRMPs, OPP, OGR, JBO and WLB) explained 56.9% of the variation in TOI. On the other hand, the variables OPP, OGR and HRMPs explain 50.7% of the variance in sustaining WLB practise, with the *R*^2^ value of (0.507).

As indicated in Table 4, the results of the path analysis revealed that OPP (β = −0.176, *p* < 0.05), OGR (β = −0.009, *p* < 0.05) and WLB (β = −0.147, *p* < 0.05) has a negative and significant effect on TOI, thus supporting H_1A_, H_1C_ and H_3_. The path value between JBO and TOI (β = 0.346, *p* < 0.05) revealed that JBO significantly and positively affected TOI, which supported H_5A_. Contrary to our prediction, the impact of the HRMPs (β = −0.019, *p* = 0.354) on TOI is insignificant; therefore, H_1B_ was rejected.

**Table 4:** Hypothesis testing direct effects.

Hypothesis	Relationship	Beta	*t* value	Value of *p*	95% BCI LL	95% BCI UL	*r* ^2^	*f* ^2^	Decision
Independent variables on EIL									
H_1A_	OPP→EIL	−0.176	3.382	0.000	−0.264	−0.093	0.569	0.029	Accepted
H_1B_	HRMP→EIL	−0.019	0.373	0.354	−0.103	0.068		0.001	Rejected
H_1C_	OGR→EIL	−0.099	1.852	0.032	−0.186	−0.012		0.007	Accepted
H_3_	WLB→EIL	−0.147	2.603	0.005	−0.239	−0.054		0.018	Accepted
H_5A_	JBO→EIL	0.346	8.806	0.001	0.28	0.409		0.192	Accepted
Independent variables on WLB									
H_2A_	OPP→WLB	0.134	2.861	0.002	0.058	0.212	0.507	0.015	Accepted
H_2B_	HRMP→WLB	0.326	6.092	0.000	0.237	0.412		0.083	Accepted
H_2C_	OGR→WLB	0.319	5.653	0.001	0.227	0.412		0.071	Accepted

OPP, occupational prestige; HRMP, human resource management practise; OGR, organisation reputation; WLB, work–life balance; JOB, job opportunity; and EIL, employee intention to leave. A 95% CI with a bootstrapping of 5,000 was used. Author’s data analysis.

Furthermore, the result of the study also provided support for the positive and significant impact of OPP (β = 0.134, *p* < 0.05), OGR (β = 0.319, *p* < 0.05) and HRMPs (β = 0.326, *p* < 0.05) on WLB, thus supporting H_2A_, H_2B_ and H_2C_.

### 4.4. Mediation analysis of WLB

We used interval biases corrected 95% to test the mediating role of WLB. The results of biases corrected 95% demonstrate that the upper and lower CI did not straddle a zero, thus confirming that WLB significantly mediated the impact of HRMPs, OPP and OGR on TOI. Thus, H_4A_, H_4B_ and H_4C_ are supported. Therefore, based on the results, it was concluded that WLB practises partially mediated the relationship between TOI, OPP and OGR and fully mediated the relationship between HRMPs and TOI (Table 5).

**Table 5:** Hypothesis testing indirect effect.

Hypothesis	Relationship	Beta	*t* value	Value of *p*	95% BCI LL	95% BCI UL	Decision
H_4A_	OPP→WLB→EIL	−0.02	0.01	1.884	0.03	−0.042	Mediation
H_4B_	HRMP→WLB→EIL	−0.048	0.02	2.347	0.009	−0.086	Mediation
H_4C_	OGR→WLB→EIL	−0.047	0.02	2.341	0.01	−0.085	Mediation

OPP, occupational prestige; HRMP, human resource management practise; OGR: organisation reputation; WLB: work–life balance; JOB, job opportunity; and EIL, employee intention to leave. A 95% CI with a bootstrapping of 5,000 was used. Author’s data analysis.

### 4.5. Moderation analysis

**Figure 2:**
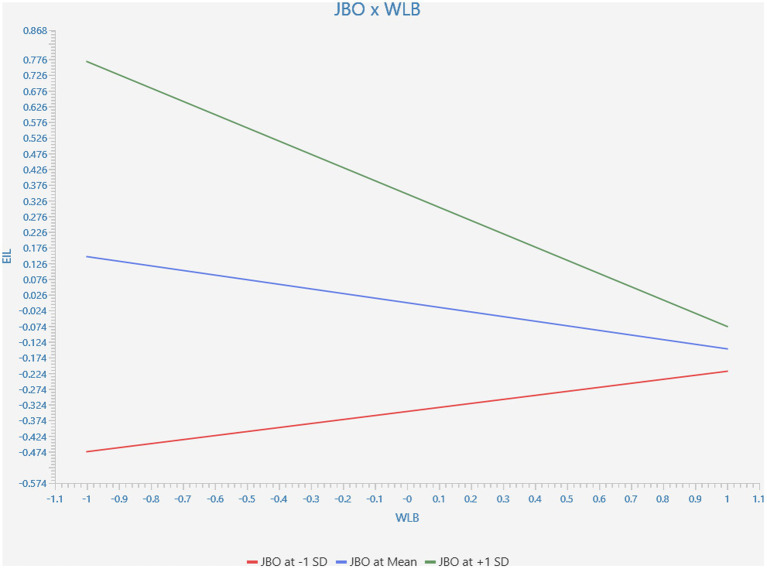
Moderating effect of job opportunity.

To test the moderating role of JBO between WLB and TI, we created an interaction term of JBO* WLB. The results obtained through bootstrapping analysis demonstrated that JBO (β = −0.278, *p* < 0.05) significantly moderated the relationship between WLB and TOI, thereby supporting H_5B_ (see Table 6; Figure 2).

**Table 6:** Moderating effects.

Hypothesis	Relationship	Beta	*t* value	Value of *p*	BCI LL	BCI UL	*f* ^2^	Decision
H_5B_	JBO × WLB- > EIL	−0.275	6.892	0.000	−0.341	−0.21	0.105	Moderation

WLB, work–life balance; JBO, job opportunity; EIL, employee intention to leave.

## 5. Discussion, implications and limitations

### 5.1. Discussion

In this study, (i) the direct effects of OPP, HRMPs and OGR on TOI, (ii) the mediating role of WLB and (iii) the moderating role of JBO on the relationship between TOI and WLB practise were empirically examined. The results indicated that OPP and OGR were directly linked to academic TOI while HRMPs were indirectly related to TOI through WLB practise. Thus, the study offered support to accept H_1A_ and H_1C_, and rejected H_1B_. The outcomes of the study are in line with the findings of Dalton et al. (2020), who illustrated that OPP significantly influenced TOI and burnout. The finding was consistent with the results by Kakar et al. (2021), who demonstrated that OGR was a negative and significant determinant of TOI. Nonetheless, HRMPs had a negative but insignificant influence on TOI, which disagreed with the result of Rashid (2021), Kakar et al. (2019), and Kakar and Saufi (2021), who indicated that HR practises significantly affected employee retention. This finding can be explained by the possibility that some employees may favourably evaluate HRMPs, which include training, while others might regard them negatively. Therefore, management must determine what HRM personnel prefer and need, where younger academicians would benefit from training and career development possibilities while senior employees would benefit from compensation and retirement plans.

Second, the analysis confirmed that WLB significantly reduced TOI, thus, supporting H3. The result was in accordance with that of Kakar et al. (2022) and Ozioma et al. (2022), who demonstrated that WLB practises negatively affected academic intention to leave. Additionally, the analysis indicated that OPP, HRMPs and OGR exerted a positive significant effect on WLB, supporting H_2A_, H_2B_ and H_2C_.

Third, JBO was established as the most influential predictor of TOI. The study offered a substance to accept H_5A_. The moderating analysis confirmed that JBO significantly moderated the relationship between TOI and WLB, thus, accepting H_5B_. The findings revealed that regardless of organisational WLB policies, employees are more likely to leave their jobs when JBOs are plentiful. Therefore, the findings supported the significance of JBO as a contextual factor and moderating variable in the turnover literature, which supported recent findings (Samad and Saufi, 2017; Kakar et al., 2019; Sepahvand and Khodashahri, 2021). Thus, JBO availability facilitated academic employees’ intention to leave their university. This study offers new perspectives on how contextual factor might either increase or weaken the link between WLB and TOI. Particularly, available jobs, which are a crucial element of mobility, might amplify the impact of WLB on TOI.

Finally, WLB practises fully mediated the association between HRS and TOI and partially mediated the relationship betweenTOI, OPP, and OGR. The finding matched the postulation by Kaur and Randhawa (2021), who illustrated that WLB mediated the relationship between employee TOI and perceived supervisor support. The study’s findings support conclusion of Suganda (2022) that WLB also functions as a mediator in the association between psychological capital and TOI.

### 5.2. Implications

#### 5.2.1. Theoretical implication

This study has made several contributions to theories and literature. First, this study has contributed to TBP and SET by extending their application in the educational industry and explaining the role of OPP, PGR and HRMPs in reducing employees’ TOI. Second, this study contributed to the literature by demonstrating the significant relationship between OPP, OGR and HRMPs and TOI. The investigation of WLB as a mediator between TOI and OPP, OGR and HRMPs, and the moderating implications of JBO between WLB and TOI in academia are two original contributions of this research.

According to the TPB and SET, employees are more likely to feel proud at work and at home if they believe they work for a highly esteemed and reputable organisation. They will consequently encounter less work–family friction, which lowers their TOI. Additionally, employees will experience WLB when an organisation invests in them by offering HRMPs that are advantageous for both their work and family lives. This perception of WLB will lower their TOI. Thus, implementing a strategy to boost WLB would therefore be successful in retaining employees when the job market is getting worse or when employees’ opinions of their employment possibilities are poor.

Past studies placed scarce attention on the mediating effect of WLB and the moderating role of JBO in the interaction between WLB and TOI. In this study, earlier research was superseded by an examination of the interplay between employee TOI, JBO and WLB. This examination is crucial given the reasons that inspire employees to explore available employment before leaving their existing job. Thus, the findings contributed significantly to the SET and TPB and clarified the JBO as a contextual factor between WLB and TOI, given that the decision to leave a job was influenced not only by WLB but also by job opportunity availability and accessibility.

#### 5.2.2. Practical implication

The findings might yield significant implications for decision-makers, specifically university authorities and HR managers. The results indicated that employees’ conceptions of OGR and OPP improved their WLB and reduced their TOI. Thus, strategies for enhancing the reputation and prestige of a university must include actions that are advantageous to how both internal stakeholders and external audiences view the societal position of an organisation. The university administrations can create, build and maintain a good reputation by ensuring that all stakeholders (employees, students, family members and the community) have positive perceptions, experiences and satisfaction with university services. Managers must guarantee that the institutions are effective and efficient so that stakeholders will disseminate positive messages regarding the organisation. Employees predominantly choose to work for organisations that support families, are socially conscious, are known for their commitment to employees’ welfare and provide a positive work environment. Thus, by sustaining WLB and providing a supportive work environment that promotes social responsibility, employee well-being and a family-friendly setting, universities may improve their reputations with stakeholders, which in turn might significantly contribute to reducing academic TOI.

Furthermore, the findings of the study imply that respecting employees’ perspectives and conferring them more decision-making authority may increase their perception of the prestige of their profession. Besides, approaching employees with respect and attentiveness and valuing their opinions and perspectives may aid the generation of a good impression of academic services. Implementing strategies to improve working conditions, reduce workloads and provide flexibility and discretion may improve a manager’s reputation in the workplace (Kakar et al., 2019). Therefore, managers should offer flexible work schedules, job-sharing and decision-making authority to personnel to improve the professional reputation of educational services.

In this study, it was determined that employees’ good attitudes towards HRMPs improved WLB, which in turn reduced their desire to leave the job. The major contribution thus focused on the direct effects of HRMPs on WLB and the intermediary role of WLB in connecting HRMPs, OGR, OPP and TOI. This mediating mechanism presented a novel insight into the relationship between employee turnover and their predictors. Moreover, the availability of future prospects, attractive salaries and training programmes would encourage academic employees to have a favourable opinion of HRMPs. Additionally, management must inform workers of organisational ‘expectations and provide them with the necessary resources. The dissemination of HRMPs could reduce misunderstandings and conflicts over the fundamental purposes and aims of these activities, which would improve academicians’ perceptions of these practises (Kakar et al., 2018). Furthermore, the administration should promote fairness, impartiality and justice by embracing and implementing moral, sensible, consistent and environmentally beneficial HRMPs and WLB strategies. By strengthening the WLB and fostering a positive image or perception of HRM policies and practises among employees, these regulations will decrease the possibility of TOI.

As WLB have a substantial mediating role in the interaction between dynamic HRMPs, OGR, OPP and TOI, they supersede the mere reduction of employees’ TOI. Therefore, the administration must offer WLB practises, which include flexible scheduling, maternity and paternity leave, health and wellness programmes and childcare facilities, to reduce academic employees’ TOIs (Lestari and Margarethaa, 2021). When employees perceive that the organisation is applying WLB practises, they might realise that they are respected and accounted for by the institution. This notion of receiving excellent care will increase employees’ workplace connection and reduce their inclination to leave in accordance with reciprocity rules.

Besides, the study’s findings have a number of implications for human resource professionals who are developing plans to control employee turnover intentions. The findings demonstrated empirically that WLB was not only negatively associated with employee intention to leave but also significantly mediated the relationship between employee intention to leave and HRMPS, OGR and OPP, which are consistent with earlier studies (Kakar et al., 2021; Aman-Ullah et al., 2022; Suganda, 2022). Furthermore, job opportunities positively moderated the relationship between WLB and the employee’s intention to leave. Adopting and promoting a strategy to improve WLB would therefore be effective in retaining employees when the labour market is getting worse or when employees’ perceptions of their employment opportunities are low. Moreover, employees’ perceptions of career opportunities aid in increasing their connection with the university, which may further result in employees’ emotional attachment with their institution, which in turn may diminish their intentions to quit the organisation.

### 5.3. Limitations

Despite the significant theoretical and managerial achievements, the cross-sectional design of this study was a drawback as it might limit the extent to which the results can be generalised. Thus, potential researchers may contribute to the knowledge corpus by placing the suggested question model in a longitudinal survey. Second, data were only gathered from one source; as a result, future research will be more valuable if information is obtained from a variety of sources, such as administrators and academicians. Second, the technique would be more beneficial if information were collected from diverse sources, such as administrators and academicians, as the data in this study was obtained only from one source. Finally, the current data was gathered from the national higher education sector and cannot be considered representative of other sectors. Therefore, the conclusions cannot be applied to employees in other businesses. Data should be gathered from employees working in organisations from various sectors in various parts of the country to provide a more accurate representation of the diverse industries.

## Data availability statement

The raw data supporting the conclusions of this article will be made available by the authors, without undue reservation.

## Ethics statement

The studies involving human participants were reviewed and approved by UMK Research ethics committee. The patients/participants provided their written informed consent to participate in this study.

## Author contributions

NC, PP, NZ, and AK focused on the conceptualisation, methodology, and resources. SA performed writing—original draft preparation. RA performed writing—review and editing, visualisation and supervision. All authors contributed to the article and approved the submitted version.

## Funding

This research was funded by the Ministry of Higher Education, Malaysia under the grant entitled “Fundamental Research Grant Scheme—FRGS” (Grant Code: FRGS/1/2020/SS02/UMK/01/1) and Dynamism of Hr Strategy Through Academic Person-environment Fit Towards Sustaining Work Life Balance and Reducing Intention to Leave.

## Conflict of interest

The authors declare that the research was conducted in the absence of any commercial or financial relationships that could be construed as a potential conflict of interest.

## Publisher’s note

All claims expressed in this article are solely those of the authors and do not necessarily represent those of their affiliated organizations, or those of the publisher, the editors and the reviewers. Any product that may be evaluated in this article, or claim that may be made by its manufacturer, is not guaranteed or endorsed by the publisher.

## Supplementary material

The Supplementary material for this article can be found online at: https://www.frontiersin.org/articles/10.3389/fpsyg.2023.1137945/full#supplementary-material

## Appendix

Appendix A1 Discriminant validity.

**Table:**

	HRMP	ILT	JBO	OGR	OPP	WLB	JBO x WLB
HRMP1	**0.911**	−0.5	−0.384	0.715	0.666	0.615	0.336
HRMP2	**0.919**	−0.51	−0.391	0.703	0.662	0.629	0.347
HRMP3	**0.92**	−0.509	−0.364	0.685	0.625	0.593	0.363
HRMP4	**0.915**	−0.497	−0.361	0.672	0.629	0.591	0.347
HRMP5	**0.908**	−0.493	−0.365	0.672	0.64	0.569	0.337
HRMP6	**0.915**	−0.497	−0.381	0.675	0.628	0.612	0.347
HRMP7	**0.904**	−0.5	−0.368	0.676	0.626	0.588	0.335
HRMP8	**0.917**	−0.51	−0.389	0.682	0.633	0.6	0.331
HRMP9	**0.893**	−0.531	−0.396	0.699	0.657	0.613	0.318
EIL1	−0.527	**0.97**	0.545	−0.543	−0.542	−0.619	−0.425
EIL2	−0.526	**0.97**	0.529	−0.532	−0.539	−0.614	−0.427
EIL3	−0.545	**0.964**	0.543	−0.549	−0.542	−0.614	−0.429
EIL4	−0.536	**0.957**	0.535	−0.546	−0.534	−0.613	−0.444
EIL5	−0.545	**0.956**	0.536	−0.551	−0.534	−0.614	−0.441
EIL6	−0.511	**0.936**	0.528	−0.526	−0.535	−0.594	−0.43
JBO1	−0.419	0.568	**0.986**	−0.412	−0.409	−0.507	−0.079
JBO2	−0.399	0.545	**0.972**	−0.383	−0.374	−0.485	−0.1
JBO3	−0.396	0.539	**0.984**	−0.393	−0.381	−0.481	−0.07
JBO4	−0.413	0.542	**0.983**	−0.403	−0.387	−0.489	−0.075
OGR1	0.725	−0.553	−0.391	**0.94**	0.698	0.647	0.326
OGR2	0.729	−0.555	−0.406	**0.943**	0.7	0.644	0.328
OGR3	0.709	−0.53	−0.381	**0.936**	0.686	0.623	0.291
OGR4	0.702	−0.508	−0.361	**0.939**	0.701	0.611	0.294
OGR5	0.707	−0.509	−0.353	**0.938**	0.695	0.611	0.278
OGR6	0.696	−0.53	−0.386	**0.942**	0.686	0.617	0.296
OGR7	0.682	−0.521	−0.385	**0.933**	0.692	0.604	0.296
OPP1	0.672	−0.53	−0.371	0.689	**0.931**	0.574	0.294
OPP2	0.595	−0.499	−0.378	0.647	**0.893**	0.533	0.193
OPP3	0.691	−0.531	−0.363	0.701	**0.937**	0.563	0.3
OPP4	0.67	−0.537	−0.37	0.687	**0.93**	0.564	0.297
OPP5	0.654	−0.519	−0.35	0.688	**0.933**	0.538	0.26
OPP6	0.657	−0.518	−0.364	0.7	**0.947**	0.579	0.276
OPP7	0.593	−0.48	−0.356	0.657	**0.88**	0.51	0.187
WLB1	0.638	−0.618	−0.48	0.631	0.576	**0.962**	0.505
WLB2	0.628	−0.602	−0.485	0.627	0.568	**0.964**	0.511
WLB3	0.651	−0.62	−0.486	0.658	0.588	**0.977**	0.501
WLB4	0.634	−0.623	−0.481	0.647	0.582	**0.962**	0.484
JBO x WLB	0.373	−0.451	−0.083	0.321	0.282	0.518	1
